# B-cell activating factor in the pathophysiology of multiple myeloma: a target for therapy?

**DOI:** 10.1038/bcj.2015.3

**Published:** 2015-02-27

**Authors:** P J Hengeveld, M J Kersten

**Affiliations:** 1 Department of Hematology, Academic Medical Center, Amsterdam, The Netherlands

## Abstract

Multiple myeloma (MM) is a currently incurable malignancy of plasma cells. Malignant myeloma cells (MMCs) are heavily dependent upon the bone marrow (BM) microenvironment for their survival. One component of this tumor microenvironment, B-Cell Activating Factor (BAFF), has been implicated as a key player in this interaction. This review discusses the role of BAFF in the pathophysiology of MM, and the potential of BAFF-inhibitory therapy for the treatment of MM. Multiple studies have shown that BAFF functions as a survival factor for MMCs. Furthermore, MMCs express several BAFF-binding receptors. Of these, only Transmembrane Activator and CAML Interactor (TACI) correlates with the MMC's capability to ligate BAFF. Additionally, the level of expression of TACI correlates with the level of the MMC's BM dependency. Ligation of BAFF receptors on MMCs causes activation of the Nuclear Factor of κ-B (NF-κB) pathway, a crucial pathway for the pathogenesis of many B-cell malignancies. Serum BAFF levels are significantly elevated in MM patients when compared to healthy controls, and correlate inversely with overall survival. BAFF signaling is thus an interesting target for the treatment of MM. Several BAFF-inhibitory drugs are currently under evaluation for the treatment of MM. These include BAFF-monoclonal antibodies (tabalumab) and antibody-drug conjugates (GSK2857916).

## Introduction

Multiple myeloma (MM) is characterized by the malignant proliferation of plasma cells, terminally differentiated B-cells which under normal circumstances are responsible for the mass production of immunoglobulins. The capability of complete or fractal immunoglobulin production is often retained in malignant myeloma cells (MMCs), resulting in the overproduction of a monoclonal protein, which can result in disease-related symptoms such as cast nephropathy and hyperviscosity. Other manifestations of MM include impaired hematopoiesis and pancytopenia, extensive skeletal destruction and hypercalcemia.

MM is the second most prevalent hematologic malignancy, with an estimated global incidence of 102 000 new cases and a global mortality of 72 000 cases yearly, which is approximately 1% of the global burden of cancer.^1^ Incidence rates range from 0.4 to 5 per 100 000, increasing markedly with age and with a male predominance.^2^ Despite recent progress in the treatment of MM, it remains an incurable condition. This underscores the need for the development of new, more effective drugs.

The progression from plasma cell to MMC is characterized by multiple oncogenic events, such as hyperdiploidy and deregulation of *cyclin D1.* Despite these genetic alterations, the malignant plasma cell remains largely dependent upon its bone marrow (BM) niche for survival. This dependency provides a rationale for targeted therapy aimed at disruption of the interaction between the MMC and the constituents of its BM microenvironment. Of particular interest is one specific humoral component of the BM microenvironment: B-cell activating factor belonging to the tumor necrosis factor (TNF) family (BAFF). This review will describe the relevance of BAFF to the physiology of humoral immunity, the role of BAFF and its receptors in the pathophysiology of MM and subsequently the potential of inhibiting BAFF signaling as a treatment option for MM will be discussed.

## MM and the BM microenvironment

**Figure 1:**
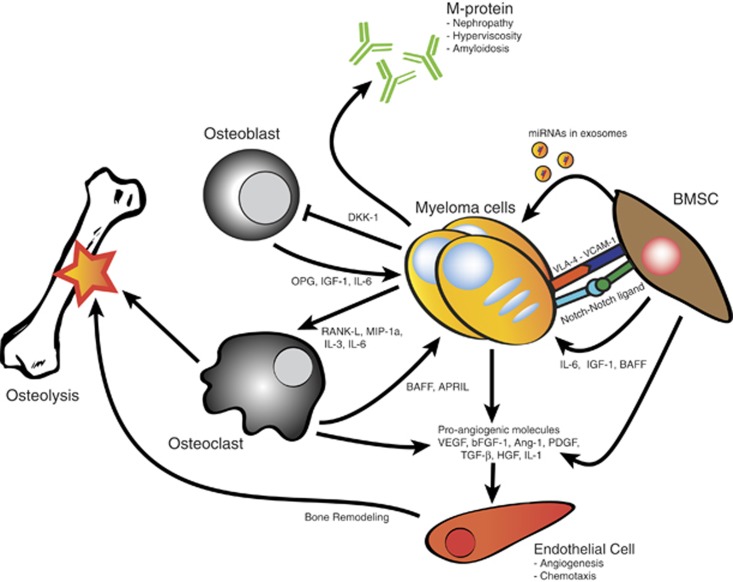
The BM micro-environment of MM. MMCs, which produce M-protein, reside in the BM and are surrounded by a variety of non-hematopoietic cells, including BMSCs, endothelial cells, osteoclasts and osteoblasts. BMSCs produce a variety of growth factors for the MMCs, and provide signaling through adhesion molecules, Notch-notch interaction and exosome transmission. Osteoclasts produce BAFF and APRIL, which are MMC growth factors, and their osteolytic activity is stimulated by cytokines produced by MMCs. Osteoblast function is inhibited by MMC produced cytokines. Additionally, osteoblasts secrete several factors which enhance MMC survival. MMCs, BMSCs and osteoclasts furthermore produce pro-angiogenic molecules, which act on the endothelial cells to stimulate angiogenesis, chemotaxis and bone remodeling.

Interaction between the constituents of the BM microenvironment and MMCs has been shown to enhance MMC differentiation, migration, proliferation and survival as well as the development of drug resistance. These pathophysiological processes arise through complex interactions between the MMC and the different cellular and extracellular components of the BM microenvironment (see Figure 1).

### Cellular component

The cellular component of the BM microenvironment encompasses BM mesenchymal stromal cells (BMSCs), endothelial cells, osteoclasts and osteoblasts. BMSCs facilitate the proliferation and survival of MMCs through adhesion, paracrine secretion,^3^ Notch signaling^4^ and the production of pro-angiogenic molecules.^5^ Furthermore, BMSCs have been shown to transfer microvesicles containing micro-RNAs to MMCs, resulting in the modulation of tumor growth *in vivo*, a process known as exosome transmission.^6^ Endothelial cells facilitate angiogenesis, either through the secretion of angiogenic factors or through the recruitment of endothelial progenitor cells to the vascular niche.^7^ Osteoclasts promote angiogenesis through the production of osteopontin,^8^ while osteoblasts have been observed to produce growth and survival factors in co-culture with MMCs.^9^

### Non-cellular component

The non-cellular component of the BM microenvironment encompasses the extracellular matrix and several humoral growth and survival factors. The humoral components of the BM microenvironment have been extensively researched because of their potential as targets for monoclonal antibody (mAb) therapy.

BAFF and APRIL, members of the TNF superfamily, are two such humoral factors involved in the pathophysiology of MM.^10^ Recently, several inhibitors of BAFF signaling have been developed for the treatment of autoimmune diseases.^11^

## BAFF in the physiology of normal B cells and plasma cells

BAFF (*TNFSF-13B*) was simultaneously discovered in 1999 by several independent research groups, under the names of BlyS,^12^ THANK-1,^13^ TALL-1^14^ and BAFF.^15^ BAFF is a 285-amino acid type-II transmembrane protein, and a member of the TNF superfamily (*TNFSF-13B*). Surface-bound BAFF can be cleaved by a furin protease, resulting in a soluble, 152-amino acid 17-kDa molecule.^15^ APRIL (synonyms TALL-2, TRDL-2, *TNFSF-13A*), another member of the TNF superfamily, exhibits a high level of sequence similarity (~30% homology) to BAFF. Soluble BAFF forms biologically active homotrimers, but can also form heterotrimers with APRIL.^16^ The relevance of the formation of these heterotrimers is unknown. Furthermore, at neutral or basic pH, 20 trimers of soluble BAFF have been observed to associate into a 60-mer virus-like particle.^17^

The BAFF gene is located at the distal arm of chromosome 13 (13q34). It consists of 6 exons, of which exon 1 encodes the transmembrane domain, exon 2 a furin processing site and exon 3-6 encodes a TNF homology domain which binds TNF receptors.^18^ Interestingly, alternative splicing leads to a short variant of the BAFF molecule (ΔBAFF). This isoform forms inactive heterotrimers with BAFF *in vivo*, functioning as a negative regulator of BAFF signaling.^19^

### Receptors of BAFF

**Figure 2:**
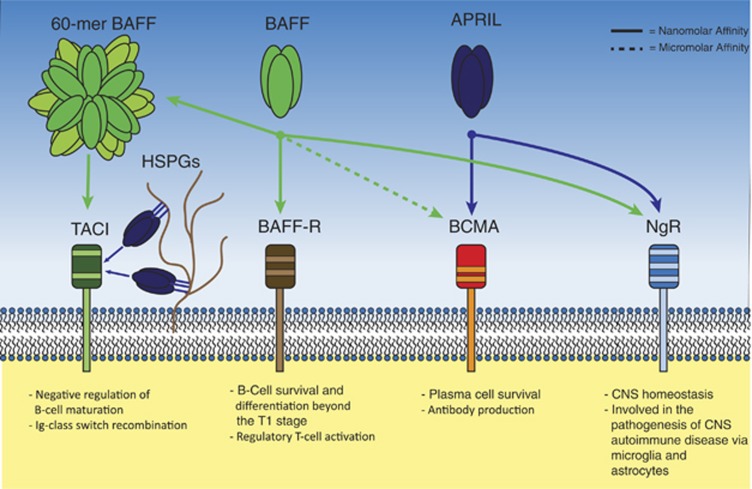
BAFF, APRIL and their receptors. Soluble BAFF and APRIL can activate multiple transmembrane receptors. The BAFF-R is a selective receptor for BAFF, and is important in early B-cell homeostasis and regulatory T-cell function. BCMA binds APRIL with nanomolar affinity and BAFF with micromolar affinity. BCMA expression is restricted to antibody-producing cells and BCMA function is paramount for plasma-cell longevity. The NgR binds both APRIL and BAFF and is important for CNS homeostasis and plays a role in CNS autoimmune diseases. TACI can bind 60-mer BAFF and heparan sulphate proteoglycan-bound APRIL with nanomolar affinity, and is implicated as negative regulator of B-cell maturation. Additionally, TACI is important for Ig-class switch recombination in the germinal center.

BAFF can bind to three receptors of the TNF receptor (TNFR) family (see Figure 2): Transmembrane Activator and Calcium Modulator and Cyclophilin ligand Interactor (TACI, *TNFRSF-13B*), B-cell Maturation Antigen (BCMA, *TNFRSF-17*) and the BAFF receptor (BAFF-R, synonyms BR3 and *TNFRSF-13C*). All three receptor subtypes are type III transmembrane proteins which lack a signal-peptide and contain cysteine-rich extracellular domains. The affinity of BAFF is in the nanomolar range for BAFF-R and TACI and in the micromolar range for BMCA. In contrast, APRIL demonstrates nanomolar affinity for BCMA and TACI, but is unable to ligate the BAFF-R.^20^ Interestingly, TACI activation requires additional multimerization such as assembly of BAFF into a 60-mer particle or oligomerization of APRIL by binding to heparan sulphate proteoglycans on the cell surface.^21^

More recently, BAFF has been implicated as a functional high-affinity ligand for the Nogo-66 receptor (NgR) on astrocytes and microglia, where engagement by BAFF is involved in the pathogenesis of several autoimmune CNS diseases.^22^

### Functions of the BAFF receptors

BAFF-deficient mice display a greater than 90% loss of B cells in all stages beyond the T1 stage,^23^ which can be compensated by the overexpression of anti-apoptotic proteins such as members of the Bcl-2 family.^24^ Comparable B-cell lymphopenia develops when mice are deficient for BAFF-R, which is normally expressed by all mature B cells.^25^ These observations prove that the BAFF/BAFF-R axis plays a vital role in the maturation and survival of B cells. Moreover, mutations leading to loss of function of BAFF-R are associated with common variable immune-deficiency disorder in humans.^26^ Interestingly, BAFF-transgenic mice develop auto-immunity and profound lymphocytosis, including a marked increase in plasma cells.^27^

BCMA-deficient mice exhibit no defect in B-cell homeostasis,^28^ but display impaired survival of long-lived plasma cells.^29^ As BCMA expression is restricted to antibody-producing cells, the BAFF/APRIL-BCMA axis is probably essential in plasma cell physiology.

The role of TACI in B-cell physiology is complex. TACI-deficient mice develop an increased number of mature B cells, have elevated serum immunoglobulin levels and display signs of systemic lupus erythematosus (SLE)-like auto-immunity, implicating TACI as a negative regulator of B-cell maturation.^30^ In contrast, *in vitro* studies with human cell lines have shown that TACI plays an important role in CD40-independent immunoglobulin class switch recombination and TACI loss of function mutations are associated with common variable immune-deficiency disorder and IgA deficiency.^31, 32^ This ambiguity suggests that TACI can serve both as a positive and as a negative regulator of B-cell differentiation, and it is speculated that the result of TACI engagement is largely context-dependent.^33^ An alternative explanation for the apparently ambivalent role of TACI could be that the absence of TACI would provide for an excess of soluble, unbound BAFF, which would be able to bind BAFF-R and thus promote B-cell longevity and auto-immunity. This would imply that TACI does not possess an intrinsic negative regulatory function.

### Production of BAFF

BAFF is expressed and secreted by several cells of the immune system, including monocytes, macrophages, dendritic cells and by a subset of T lymphocytes.^12, 13, 14, 15^ The expression of BAFF can be increased by several cytokines such as interferon-γ, interleukin (IL)-10 and granulocyte colony-stimulating factor, and by the activation of Toll-like receptors.^34, 35^ When stimulated by pro-inflammatory cytokines, BAFF expression supports ongoing immune responses and stimulates the activation of the humoral immune system. Additionally, a significant fraction of circulating BAFF is produced by non-myeloid radiation-resistant cells, which are most likely to be stromal cells such as osteoclasts.^36^

## The role of BAFF in the pathophysiology of MM

Both human myeloma cell lines (HMCLs) and primary MMCs are highly capable of binding soluble BAFF^10, 37, 38, 39, 40, 41, 42^ and, when cultured with recombinant human BAFF, show enhanced survival, proliferation, long-term growth and resistance to dexamethasone and lenalidomide,^10, 41, 42, 43, 44, 45, 46^ comparable with the effects of the established MMC growth factor IL-6.^43^ Additionally, the elimination of BAFF with a BAFF-mAb in a human myeloma mouse model resulted in a decrease of tumor burden, protected against lytic bone disease through decreased osteoclastogenesis and led to an overall increase in survival.^44^ These observations emphasize the importance of BAFF in the pathogenesis of MM.

### BAFF receptors in MM

BCMA is invariably expressed in HMCLs and in most primary MMCs,^10, 38, 39, 41, 42, 43, 44^ and comparative gene expression profiling has shown increased expression of BCMA in MMCs when compared with normal plasma cells.^44, 47, 48^ Furthermore, successful donor lymphocyte infusion in MM patients is associated with the development of anti-BCMA antibodies, suggesting the presence of BCMA on the cell-surface of MMCs.^49^ However, despite its invariable presence, BCMA expression does not correlate with the capability to ligate BAFF, possibly because of the 1000-fold weaker affinity of BAFF for BCMA than for BAFF-R or TACI.^42^

BAFF-R expression is absent in most HMCLs,^38, 50^ but has been reported to be variably present in primary MMCs,^10, 38, 40, 41, 43, 44, 45, 50, 51, 52, 53^ albeit at significantly lower levels than the expression of both BCMA and TACI.^43^ The discrepancy between the expression of BAFF-R in HMCLs and primary MMCs could be explained by the fact that HCMLs are often cultured from an extramedullary MMC in late stage MM. The loss of BM dependency in these cells might have permitted the downregulation of BAFF-R.^42^

TACI expression is variable in both HMCLs and MMCs,^10, 38, 39, 40, 41, 42, 43, 44, 54^ but it is indicative of the capability to ligate BAFF^39, 42^ and expressed at high levels in gene expression profiling studies.^44, 47, 48^ Moreover, gene expression profiling studies have shown that MMCs with high TACI expression share a high degree of genetic similarity with long-lived, BM-dependent plasma cells, whereas MMCs with low TACI expression cluster with polyclonal plasmablasts, which display low BM dependency. This observation suggests that a shift in TACI expression and the capability to ligate BAFF is indicative for the progression to extramedullary proliferation of MM.

In conclusion, the above data imply an essential role for TACI in MM-associated BAFF signaling, but a role for neither BAFF-R nor BCMA can be excluded.

### BAFF signaling cascade in MM

Ligation of BAFF to its receptor can activate either the canonical or non-canonical NF-κB pathways, resulting in the nuclear translocation of p50/p65 and/or p52/RelB and the subsequent upregulation of anti-apoptotic proteins, most notably Bcl-2, Bcl-xL, Bcl-w, Mcl-1 and A1, and downregulation of pro-apoptotic proteins, most notably Bid, Bad, Bik, Bim, Bmf, Hrk/DP5, Noxa and Puma, and the exclusion of PKCδ from the nucleus.^10, 41, 43, 45, 55^ This process is vital for the survival of many B-cell malignancies, including MM.^56^

**Figure 3:**
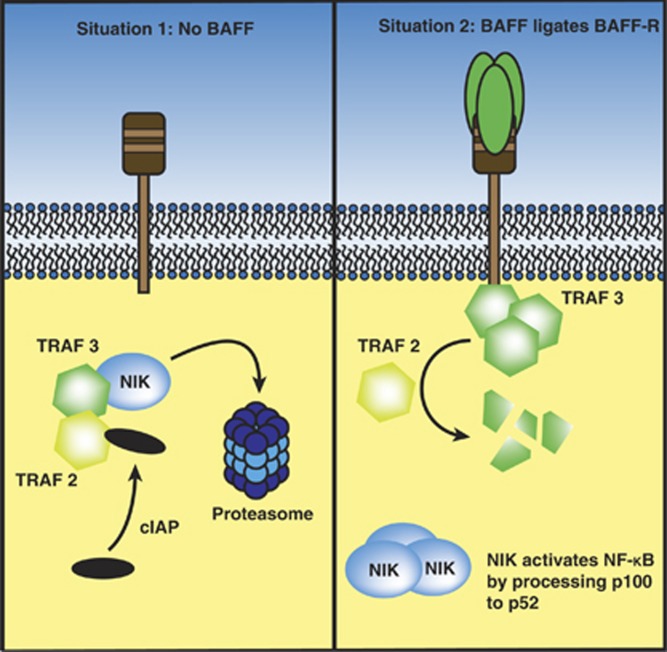
Downstream signaling of BAFF-R. Situation 1: When the BAFF-R remains inactivated, NIK forms a complex with TRAF3 and TRAF2. TRAF2 then recruits cIAP-1 and -2 to the NIK-TRAF complex. The cIAPs then target NIK for proteasomal degradation, hereby inhibiting the activation of the alternative NF-κB pathway. Situation 2: Activation of the BAFF-R by BAFF recruits TRAF3 to the cytoplasmic tail of the receptor, after which TRAF2 mediates the degradation of TRAF3. NIK accumulates and is now free to process p100 to p52, initiating the activation of genes associated with cell survival. Inactivating mutations of either TRAF2, TRAF3 and cIAP genes have been found in the majority of BM-independent HMCLs.

Interestingly, up to 30% of HCMLs exhibit genetic aberrations leading to the constitutive activation of NF-κB signaling.^57^ Frequently, these mutations affect BAFF-associated pathways: more than 50% of these aberrations cause loss-of-function of *traf3*, which codes for TNF-receptor associated factor (TRAF)-3, a signaling protein of the intracellular pathway of BAFF-R and a negative regulator of non-canonical NF-κB activation.^58^ Additionally, other proteins of the BAFF-R signaling cascade display aberrations in HMCLs, such as NF-κB inducing kinase (NIK), cellular inhibitor of apoptosis (cIAP-)1/2 and TRAF2.^56^ For an overview of how these molecules interact in the downstream signaling of BAFF, see Figure 3. Furthermore, HMCLs incidentally display gain-of-function mutations of TACI.^56^ All these mutations in HMCLs, which resemble extramedullary MMCs, affect BAFF-associated pathways and cause constitutive activation of NF-κB.

These data support the hypothesis that MMCs residing in the BM, which lack the aforementioned mutations, are highly dependent on external BAFF signaling for the activation of NF-κB and survival. Additional to the activation of NF-κB, BAFF can activate MAP-kinase pathways such as c-Jun NH_2_ terminal kinase (JNK) and the phosphatidylinositol (PtdIns) 3-kinase (PI3K) pathway. The activation of both these pathways results in prolonged survival and increased proliferation.^44, 46^

### BAFF production in MM

BAFF signaling is hypothesized to function in both an autocrine and paracrine manner. Several studies show that MMCs are capable of the production, expression and secretion of BAFF,^10, 37, 38, 40, 41, 43, 46, 59^ implying an autocrine signaling system. However, Moreaux *et al.*^39^ showed that the BAFF production by the BM microenvironment, in particular by polymorphonuclear cells and osteoclasts, is 100-fold higher than the BAFF production by purified MMCs. This establishes that BAFF signaling acts primarily through a paracrine mechanism, with a minority of the MMC population capable of autocrine BAFF production, thus enabling them to escape from their BM dependency. This mechanism concurs with the loops established for other humoral growth factors in MM such as IL-6.^60^

### Clinical aspects of BAFF

MM patients have a three to fivefold increase of circulating BAFF when compared with healthy controls and BAFF serum levels correlate positively with disease stage according to the International Staging System.^10, 40, 44, 61, 62, 63, 64, 65, 66, 67^ Furthermore, BAFF serum levels correlate positively with a diverse spectrum of established progression markers in MM including several ILs (IL-6, -10 and -15), lactic dehydrogenase, C-reactive protein and beta-2-microglobulin. Additionally, BAFF serum levels correlate positively with markers for angiogenesis (TNF-α, VEGF, microvessel density),^63, 64^ BM infiltration^38, 66^ and proliferation (proliferating cell nuclear antigen and Ki-67 staining).^62, 66^ Of further interest, several authors have established an inverse correlation between circulating BAFF and progression-free survival^65^ and overall survival.^62, 63^ These findings clearly establish a clinically relevant link between BAFF and MM and have prompted multiple authors to suggest BAFF as a possible biomarker for tumor burden, disease progression and prognosis and as a potential target for therapy.

## Therapies targeting BAFF signaling

### Proteasome inhibitors and immunomodulatory drugs

Recent progress in the understanding of MMC physiology led to the development of proteasome sub-unit inhibitors, such as bortezomib. By reversibly inhibiting the chymotryptic activity of the 26 sub-unit proteasome, bortezomib inhibits the NF-κB pathway, thus inhibiting proliferation and inducing apoptosis. When exposed to bortezomib, cultured MMCs show a decrease in autocrine production of both BAFF and APRIL. Furthermore, through inhibition of both the canonical and non-canonical NF-κB pathways, bortezomib interferes with the downstream signaling of BAFF and APRIL.^41^ This shows us that, although bortezomib targets multiple processes, at least a part of its anti-myeloma effect may be attributed to the inhibition of the BAFF/APRIL axis.

Immunomodulatory drugs (IMiDs), such as thalidomide or lenalidomide, have also been approved for the treatment of MM. IMiDs have multiple mechanisms of action, including the induction of direct cytotoxicity in MMCs and the inhibition of angiogenesis and osteoclasts. In the latter cells, lenalidomide inhibits the paracrine production of BAFF and APRIL.^68^ Thus, as with bortezomib, there is evidence that at least part of the anti-myeloma effect of IMiDs may be attributed to the inhibition of BAFF/APRIL signaling.

### Atacicept

Atacicept, produced by *ZymoGenetics* and *Merck Serono*, is a recombinant fusion protein that consists of the fragment crystallizable (Fc) region of human IgG and the binding domain of the TACI receptor. Atacicept is designed to bind and inactivate both BAFF and APRIL in their soluble form and thereby to inhibit their signaling.^69^ Because TACI expression on MMCs correlates directly with BM dependency, there is a strong rationale for the use of atacicept in MM. In a human myeloma mouse model, atacicept successfully showed anti-MMC activity.^70^ Similar effects were observed for a BAFFR-Ig fusion protein, albeit to a lesser extent. Further testing in co-cultured MMCs and osteoclasts showed that atacicept decreased the survival rate of MMCs and that the drug was especially effective against TACI^HIGH^ MMCs.^70^ A phase I study of atacicept in relapsed or refractory patients with MM confirmed clinical efficacy of atacicept, showing a stabilization of disease in several patients, accompanied by a stabilization of M-protein and a stabilization and/or decrease of the amount of CD138+ cells in the BM.^71^ However, no partial or complete responses were observed.

Through simultaneous neutralization of BAFF and APRIL, atacicept could give rise to clinically relevant immunosuppression. Indeed, one clinical trial of atacicept in SLE, in combined regiments with mycophenolate mofetil, was prematurely terminated owing to an increase in infectious diseases (ClinicalTrial.gov identifier: NCT00573157). This observation has prompted stagnation in the further development of atacicept for the treatment of MM and warrants caution and close monitoring of adverse events in any future clinical trials.

### Antibody drug conjugates targeting BCMA

Soluble BCMA is elevated in the serum of MM patients,^72^ and successful donor lymphocyte infusion is associated with the formation of antibodies targeting BCMA.^49^ These observations, along with the ubiquitous yet selective expression of BCMA on MMCs, provide a strong rationale for the development of mAbs targeting BCMA. Indeed, an anti-BCMA mAb, bearing triple Fc mutations (S293D:A330L:I332E) to increase the antibody-dependent cellular cytotoxicity and conjugated with a potent cytotoxic agent, monomethyl auristatin F, was tested in MM. This antibody drug conjugate displayed remarkable biological activity against MMCs in both a mono-culture and a co-culture with osteoclasts, while retaining exquisite selectivity.^73^ Tai *et al.*,^74^ in cooperation with GlaxoSmithKline, further developed this concept and produced GSK2857916, a similar anti-BCMA and monomethyl auristatin F antibody drug conjugate. This antibody drug conjugate does not bear the Fc point mutations, which can potentially cause instability and increased immunogenicity. Alternatively, the Fc glycans of GSK2857916 were defucosylated, which ameliorates the antibody-dependent cellular cytotoxicity reaction and negates the aforementioned side effects. *In vitro,* GSK2857916 displayed potent anti-MMC activity, increasing G_2_/M-arrest, apoptosis and antibody-dependent cellular cytotoxicity while leaving co-cultured BMSCs, natural killer cells and PBMCs unaffected. Further testing showed that a mere two infusions with GSK2857916 achieved complete eradication of tumor burden in a xenograft mouse model. Importantly, GSK2857916 was highly effective in a disseminated MM mouse model, which implies it could be a potential option for late-stage extramedullary MM. GSK2857916 is currently in a phase I clinical trial in MM patients (ClinicalTrial.gov identifier: NCT02064387).

Another immune-based therapy targeting BCMA is adoptive T-cell therapy. This therapy employs genetically modified T cells with a chimeric antigen receptor capable of binding BCMA. This chimeric antigen receptor-T-cell therapy showed promising anti-MM activity in *in vitro* and xenograft mouse models.^75^ Adoptive T-cell therapy could, however, in theory, produce a lasting immune reaction against BCMA-positive cells. This could, in the long term, severely compromise the healthy plasma cell population and the humoral immune system, necessitating life-long immunoglobulin suppletion. Extensive monitoring of the development of such a reaction during clinical testing is warranted.

### Direct inhibitors of BAFF

Recent progress in the field of rheumatology, sparked by the observation that BAFF serum levels are elevated in multiple auto-immune diseases such as SLE,^76^ has led to the development of several direct inhibitors of BAFF. In a phase III randomized controlled trial, belimumab, a fully human mAb against BAFF, has been shown to have a modest effect in patients with active SLE.^11^ This has led to the regulatory approval of belimumab for the treatment of SLE by the United States Food and Drug Administration (FDA) and the European Medicines Agency (EMA). Another direct inhibitor of BAFF is blisibimod, a fusion protein of four BAFF-binding peptides and the Fc locus of IgG, termed a ‘peptibody'. Blisibimod, contrary to BAFF, inhibits the action of both soluble and membrane-bound BAFF. Blisibimod is currently under evaluation in a phase III clinical trial for the treatment of SLE (ClinicalTrial.gov identifier: NCT01395745). Both belimumab and blisibimod have not yet been tested in myeloma models or patients.

Recently, Ely Lilly and Company created a selective fully human IgG4 mAb, tabalumab (LY2127399), with neutralizing activity against both membrane-bound and soluble BAFF.^77^ In murine xenograft models, tabalumab showed an anti-MM effect and inhibits osteoclastogenesis.^44^ In a phase I study, tabalumab was evaluated combined with bortezomib in relapsed or refractory MM patients. It was well tolerated and capable of inducing a partial response or better in 22/48 patients.^78^ Tabalumab is currently undergoing two additional phase I studies (ClinicalTrial.gov identifiers: NCT00689507 and NCT01556438) and a phase II/III trial (ClinicalTrial.gov: NCT01602224) in patients with MM.

**Figure 4:**
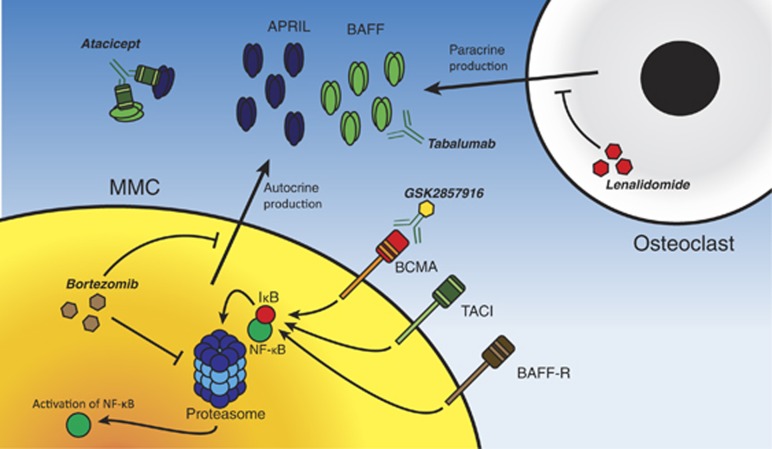
Drugs inhibiting BAFF-signaling. Bortezomib inhibits the function of the 26S proteasome. This interferes with the downstream signaling of BAFF by preventing NF-κB activation. Additionally, bortezomib inhibits the autocrine production of BAFF. Lenalidomide, an IMiD, targets the osteoclast and inhibits its paracrine production of BAFF and APRIL. New, selective inhibitory drugs are tabalumab, atacicept and GSK2857916. Tabalumab is a monoclonal antibody which targets soluble and membrane-bound BAFF. GSK2857916 is an anti-BCMA and monomethyl auristatin F antibody drug conjugate. Atacicept is a fusion protein of the Fc locus of IgG and the binding domain of TACI. It can efficiently bind APRIL and BAFF and thus inhibit their signaling.

For an illustration of the mechanism of action of aforementioned drugs, see Figure 4.

## Discussion

In this review, we have discussed the relevance of BAFF in the physiology of humoral immunity and the role of BAFF and its receptors in the pathophysiology of MM. Furthermore, we have described the progress of the development of inhibitors of BAFF signaling for the treatment of MM.

### Limitations of BAFF-inhibitory drugs

There are a few possible limitations to the use of BAFF-inhibitory therapy. Firstly, BAFF is one of the numerous growth factors in MM, and the effects of BAFF inhibition could be counteracted through alterations in other signaling pathways. Secondly, TACI is, as previously mentioned, probably the most important receptor for BAFF in MM. However, as both APRIL and BAFF share nanomolar affinity for TACI, one could speculate that the elimination of only BAFF and not APRIL might not suffice in inhibiting NF-κB activation. For this reason, drugs that inhibit both BAFF and APRIL are assumed to have a greater anti-MM effect than BAFF inhibition alone. As there have been no comparative studies between atacicept and BAFF-specific inhibitors, there is no evidence as of yet to support this assumption. Moreover, one clinical trial (ClinicalTrial.gov identifier: NCT00573157) with atacicept was terminated prematurely owing to an increased incidence of grade III infections in the group treated with atacicept. This is can probably be attributed to the fact that normal plasma cells require either BAFF or APRIL to survive, whereas atacicept blocks both. So, although atacicept is speculated to be more efficacious than BAFF inhibition alone, its use is accompanied by more severe immunosuppression.

A third limitation of BAFF-inhibitory therapy is the possible development of drug resistance. Because of the existence of subclones of MMCs already at the time of diagnosis and their subsequent clonal selection under pressure by treatment, patients with MM often develop drug resistance. As BAFF-inhibitory therapy targets external signals, it is probable that MMCs treated with this kind of therapy will develop mutations that replace the need for external BAFF stimulation, and that these subclones will be positively selected. Indeed, 30% of HMCLs, which resemble late-stage MMCs, exhibit mutational constitutive activation of NF-κB, of which more than half is comprised of mutations in BAFF signaling pathways.^57^ Possible methods to overcome the formation of resistance could involve the combination of BAFF inhibitors with existing drugs such as proteasome inhibitors or IMiDs. Alternatively, the use of BAFF-inhibitory drugs could be confined to very early stage MM or even monoclonal gammopathy of undetermined significance, which are both characterized by extensive BM dependency.

### The role of APRIL

APRIL, when bound to heparan sulphate proteoglycans, is able to ligate TACI and activate NF-κB signaling. MMCs are capable of ligating APRIL in a manner similar to BAFF, and thereby inducing increased MMC survival, proliferation and resistance to dexamethasone.^10^ Moreover, serum APRIL levels are significantly elevated in patients with MM.^64, 65^ This provides a rationale for the development of APRIL inhibitory drugs. Indeed, as mentioned before, several anti-MM drugs, including proteasome inhibitors, IMiDs and atacicept, partly achieve their effect through the neutralization of APRIL.

In contrast to the crucial role of BAFF in early B-cell homeostasis, APRIL-deficient mice only exhibit impaired IgA class-switch recombination.^79^ For this reason, APRIL-inhibitory drugs should be less likely to cause side effects due to the disruption of B-cell homeostasis than BAFF-inhibitory drugs would. Indeed, Guadagnoli *et al.*^80^ developed hAPRIL.01A, an APRIL antagonistic mAb. Testing in models of B-cell malignancies is currently ongoing.

## Conclusion

This review has evaluated the connection between BAFF and MM. Paracrine BAFF signaling, primarily through the TACI receptor, is a vital factor in the pathogenesis of early-stage BM-dependent MM. BAFF-inhibitory drugs, developed for use in auto-immune diseases, have potential benefit for the treatment of MM. Multiple BAFF-inhibitory drugs are currently in phase I or II for clinical evaluation in MM.
